# Protein recovery as a resource from waste specifically via membrane technology—from waste to wonder

**DOI:** 10.1007/s11356-020-12290-x

**Published:** 2021-01-13

**Authors:** Kanwal Shahid, Varsha Srivastava, Mika Sillanpää

**Affiliations:** 1 Department of Separation Science, School of Engineering Science, Lappeenranta-Lahti University of Technology, Sammonkatu 12, FI-50130 Mikkeli, Finland; 2 Department of Chemistry, University of Jyväskylä, P.O. Box 35, FI-40014 Jyväskylä, Finland; 3 Institute of Research and Development, Duy Tan University, Da Nang, 550000 Vietnam; 4 Faculty of Environment and Chemical Engineering, Duy Tan University, Da Nang, 550000 Vietnam; 5 School of Civil Engineering and Surveying, Faculty of Health, Engineering and Sciences, University of Southern Queensland, West Street, Toowoomba, QLD 4350 Australia; 6 Department of Chemical Engineering, School of Mining, Metallurgy and Chemical Engineering, University of Johannesburg, P. O. Box 17011, Doornfontein, 2028 South Africa

**Keywords:** Purple phototrophic bacteria, Wastewaters, Microalgae, Mesoporous silica nanoparticles, Potato processing waste, Alfalfa processing waste, Dairy waste protein, Membrane fouling

## Abstract

Economic growth and the rapid increase in the world population has led to a greater need for natural resources, which in turn, has put pressure on said resources along with the environment. Water, food, and energy, among other resources, pose a huge challenge. Numerous essential resources, including organic substances and valuable nutrients, can be found in wastewater, and these could be recovered with efficient technologies. Protein recovery from waste streams can provide an alternative resource that could be utilized as animal feed. Membrane separation, adsorption, and microbe-assisted protein recovery have been proposed as technologies that could be used for the aforementioned protein recovery. This present study focuses on the applicability of different technologies for protein recovery from different wastewaters. Membrane technology has been proven to be efficient for the effective concentration of proteins from waste sources. The main emphasis of the present short communication is to explore the possible strategies that could be utilized to recover or restore proteins from different wastewater sources. The presented study emphasizes the applicability of the recovery of proteins from various waste sources using membranes and the combination of the membrane process. Future research should focus on novel technologies that can help in the efficient extraction of these high-value compounds from wastes. Lastly, this short communication will evaluate the possibility of integrating membrane technology. This study will discuss the important proteins present in different industrial waste streams, such as those of potatoes, poultry, dairy, seafood and alfalfa, and the possible state of the art technologies for the recovery of these valuable proteins from the wastewater.

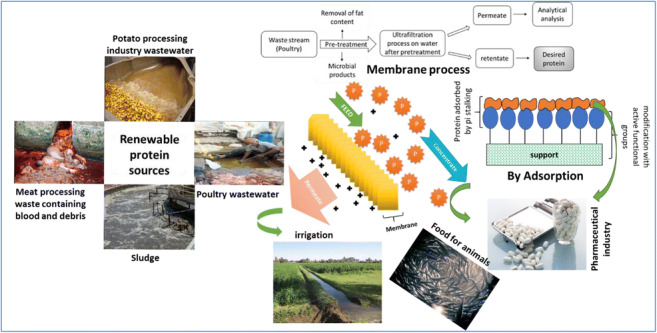

## Introduction

The most important driving force for recovering nutrients from wastewater during the treatment is the increasingly limited accessibility of resources. Population growth, fertilizer use, agricultural restrictions, soil erosion, and extreme weather events have had drastic effects, including malnutrition resulting from protein shortage. Globally, protein for animal feed is not sufficiently available; in order to meet this demand, many countries rely on the import of feed protein. At present, soybean protein (imported from other countries) serves as the main share of protein nourishment in animal feeding in Europe (Yin et al. 2011). A shortage of protein can be overcome through the optimisation of local protein resources based on the elevation of biomass concentration, especially from green plants. Inherent nutrient loss usually occurs in the upscaling of plant proteins to animal proteins, leading to the revival of the traditional idea of upgrading the manufacturing of microbial protein. The extraction of proteins from wastewater along with other resources could be an attractive low-cost alternative (Van Der Hoek et al. 2016). The wastewaters of several industries, including leather processing, contain significant concentrations of important proteins such as albumins and globulins. Furthermore, the meat processing industry together with sheep, cattle and poultry processing factories are also known to release high amounts of proteins into wastewater (Jayathilakan et al. 2012). The recovery and removal of proteins from wastewater has advantages, as these proteins are in demand for the manufacture of medicines and food additives. Such a procedure would also purify the wastewater for later use. The main issue is not the availability of resources and technology, but rather the deficiency in properly devising a structural plan and methodology to recognize the most balanced solution in a certain geographical framework (Guest et al. 2009). Various concepts have been implemented for the recovery of proteins from a waste stream, such as single-cell protein (SCP), purple phototrophic bacteria (PPB) (Meng et al. 2018) and microalgae culture (Hülsen et al. 2018). Recent research has been focused on finding the potential sources for protein recovery from sludge, grass and the wastewater of various industries. Membrane technology and adsorption have been studied for protein recovery from wastewater (Chen et al. 2007; Dabestani et al. 2017), but there is still a need to develop an efficient way to recover proteins from the waste stream. An overview of potential sources of proteins and their recovery approaches are discussed in the present study, which also gives a summary of the advances in upgrading protein waste to produce valuable materials that are prominent on technological platforms.

## Protein recovery by different approaches

### Protein recovery concept as single-cell protein (SCP)

The concept of the production of SCP is well-known, and it is a feasible way of rapidly reducing the environmental footprint. As large amounts of protein and a wide range of substrates can be converted into it (Matassa et al. 2015), SCP is therefore considered the most efficient way of recovering nitrogen from waste sources using heterotrophic bacteria and algae. SCP also has the potential to enable the bio-based circular economy. With this method, the invested nitrogen can be restored from the environment and accumulated in the form of microbial protein from a waste medium (Shi et al. 2007; Hülsen et al. 2014). SCP is hindered by its production cost, which needs to decrease in order to enable practical implementation and compete with agricultural alternatives. The main cost input in SCP production is the carbon source, but this can be minimized by using industrial waste streams such as flue gas. Due to the production of SCP, the organic contents present in the wastewater are recycled through assimilative segregation despite being lost or consumed as carbon dioxide and nitrogen gas into the air (Batstone et al. 2015).

Many researchers have tried to recover proteins using different waste media (water from several sources). The potential of purple phototrophic bacteria (PPB) and microalgae to treat diverse waste streams from agricultural and industrial sources was investigated by (Hülsen et al. 2018). PPB and microalgae are two important intermediates in the production of SCP. Microalgae are responsible for manufacturing protein-rich feed substitutes for both humans and animals (Sangameshwar Barnes et al. 2013). In the case of PPB, the utilization of infrared light has been more efficient in the production of high yields (Hülsen et al. 2014). Several researchers have focused on the removal of COD with PPB from synthetic, sterilized, and diluted wastewaters, but little is known about real wastewaters (Nagadomi et al. 2000). It can be concluded that until now there has been little focus on the removal of COD, nitrogen and phosphorus from industrial waste streams along with SCP recovery. In a recent study, diverse varieties of both PPB and algae were used to treat actual industrial and agricultural wastewaters. The important comparisons were taken into considerations that include the composite concurrent non-destructive simultaneous assimilation of COD along with nitrogen and phosphorus. The achieved yield of SCP was then estimated. The obtained discoveries can be applied to understand the possible manufacturing of single-cell proteins in our prime trades, with a focus on the newly evolving association between nutrients, energy, water and the environment (Hülsen et al. 2018).

The treatment efficiency of different industrial wastewaters with PPB and microalgae was determined by measuring parameters such as soluble COD, the concentration of ammonium and phosphate, elimination of nitrogen and total chemical oxygen demand to evaluate the removal abilities. For example, in the case of poultry wastewater treatment, PPB actively removed COD (in soluble form) along with ammonium and phosphate in a ratio of 100: 11: 1.6, respectively. The performance of PPB for the treatment of pork flesh and dairy wastewaters was reasonable but overcoming losses of organic matter is a question that still needs to be taken into consideration. However, the wastewater from the sugar industry had a higher COD value. Treating this by microalgae and PPB proved to be noxious as negligible removal of soluble amounts of COD, NH_4_-N or PO_4_-P was observed. Nevertheless, the author concluded that the microalgae tests achieved better removal efficiency of SCOD, NH_4_-N and PO_4_-P overall than PPB. The most satisfactory results were obtained with poultry wastewater treatment with 91 ± 18% COD removal, along with 91 ± 29% and 73 ± 27% of nitrogen and phosphorus removal, respectively (Khosravi-Darani et al. 2013).

The researcher also tried to characterize the biomass formed in the treatment of different waste streams. It was estimated that PPB biomass had an elevated protein content (based on solid Total Kjeldahl Nitrogen (TKN)) (Eding et al. 2006). The concentration of protein was estimated with the bicinchoninic acid assay (Ras et al. 2008). The crude protein content obtained in the present study corresponds well with other reported inquiries (Ponsano et al. 2004). The large harvest protein shows the integration of organics and nitrogen without any dissimilation (Kim et al. 2005). It was concluded that PPB and microalgae can serve as a responsible mediator for upgrading and recycling the nutrients from wastewater (Hülsen et al. 2018). A study related to the recovery of resources from wastewater was published in order to understand the production capabilities of hydrogen-oxidizing bacteria (HOBs) (Matassa et al. 2015). It covers the recovery of nutrients from wastewaters and the use of the recovered products as a starting material for the synthesis of valued biomaterials. It also elaborates on the competence of hydrogen-oxidizing bacteria to elevate the raw nitrogen and minerals into a significant microbial product. Mutually isolated and mixed diverse microbial cultures can be utilized for the tailored synthesis of restored biological compounds into complex biomolecules. It is already known that HOBs are believed to be the utmost influential contributors to the overview of biorefineries. Hydrogen-oxidizing or Knallgas bacteria (so called as they utilize gaseous hydrogen and oxygen) are autotrophic bacteria. This prominent feature gives these microorganisms numerous dietary competencies over others, including the ability to survive in a solely inorganic environment along with reducing nitrogen into new cells and more conversion of carbon dioxide (Repaske and Mayer 1976). Innovative tactics using these bacteria may have the potential to improve the nutrients restored from anaerobic digestion and waters rejected in water treatment plants during the carbon dioxide fixation process and enhance conversion activity towards biomethane. Although a sustainable and effective alternative to photosynthetic biomass construction, HOBs are viewed as a possible supplier of the microbial product in the form of SCP. The feasibility of HOBs as SCP manufacturers was later explored in a report on the properties of the proteins created by the microbes (Volova and Barashkov 2010). The biological value of proteins made by three strains of HOBs was evaluated. These strains are as follows: *Alcaligenes eutrophus Z1*, *Ralstonia eutropha B5786,* and the carbon monoxide resistant strain *carboxydobacterium Seliberia carboxydohydrogena Z1062*. The work indicated a massive amount of protein synthesized by the above-mentioned species, along with the sequences of essential amino acids. Certainly, the amino acids are more likely those of yeast, but, at about 70%, the ultimate concentration of protein, also known as dry weight in the case of hydrogen-oxidizing bacteria, is much higher than that of other species (50% in the case of yeast) (Anupama 2000).

### Protein recovery by the advance one-step photosynthetic bacteria (PSB) method

Researchers established a method known as the one-step photosynthetic bacteria (PSB) process to efficiently remove pollutants and recover nutrients from high-COD, non-toxic wastewaters (Meng et al. 2018). PSB is well-known as a collection of bacteria that can harvest light energy to pursue both the autotrophic and heterotrophic processes (Cao et al. 2020). Being state-of-the-art, PSB can efficiently treat wastewater such as that from the fish industry (Azad et al. 2004), wastewater containing starch (Getha et al. 1998; Prachanurak et al. 2014), dairy industry wastewater (Kaewsuk et al. 2010), rubber manufacturing industry wastewater, livestock waste streams (Ponsano et al. 2008), and domestic waste streams (Nagadomi et al. 2000; Hülsen et al. 2018). The possible elimination of COD (up to 85–93%) and ammonia nitrogen removal (99%) is likely to be obtained (Saejung and Thammaratana 2016; Yang et al. 2017). PSB biomass is a sound source of single-cell protein that can act as a feed for sealife. This high-grade feed effectively promotes body growth, along with an improvement in disease tolerance, with its advanced water value. PSB cells also contain carotenoids and coenzyme Q10 (CoQ10) (Hao et al. 2017). Unlike algae technology, PSB can handle wastewater with low nitrogen and phosphorous, but they cannot cope with wastewater with a high COD value. PSB technology is better in treating wastewater with a high COD content, such as the wastewater obtained from starch processing companies (potato, corn and wheat), which has a COD value of approx. 10,000 mg/L. However, PSB technology has the potential to treat this water along with a biomass harvest of up to 0.51 mg of biomass per milligramme of biological oxygen demand removal (Prachanurak et al. 2014). Different strains of PSB, additives, and different valuable substances were used to examine biomass growth. There was an attempt to use synthetic wastewater that could mimic characteristics such as COD, total nitrogen, and phosphorus concentration in brewery wastewater. The estimated measured qualities of wastewater are as follows: COD (2200-2600), total nitrogen (20–22), and total phosphorus (5–6 mg/L) (Meng et al. 2018). The characteristics of wastewater, such as the removal efficiency of the COD, total nitrogen and phosphorus (TP) and production of biomass as the value of observance with time duration using the PSB strain, are shown in Table 1 (Chen et al. 2020).

**Table 1:** Characteristics of wastewater (chemical oxygen demand, total nitrogen, total phosphorus) and production of biomass as the value of observance with time duration using the PSB strain (Meng et al. 2018; Chen et al. 2020)

Time (h)	Chemical oxygen demand (mg/L)	Total nitrogen (mg/L)	Total phosphorus (mg/L)	Total Biomass (OD_660_)
0	~2200	~17	~5	~0.3
24	~1400	~3.8	~4.7	~0.5
48	~1000	~3	~4.3	~0.62
72	800	~2.5	~4.1	~0.78

As the preliminary COD concentration of the brewery wastewater was approx. 2200 mg/L, it can be observed from Table 1 that PSB could successfully reduce the chemical oxygen demand of a given waste stream by up to 800 mg/L after 72 h along with a significant reduction in the concentration of nitrogen, but it is less effective for the removal of phosphorus. The biomass increased over time and has its applications, such as active use as a fertilizer. This method is understood to be effective for concurrent nutrient recovery and waste treatment. However, as the final COD was still very high at 800 mg/L, it was outside the stated disposal range of 80 mg/L. There is, therefore, a need for more intense research to expand COD removal with treatment by the PSB strain. The researcher tried yeast extract as a distinctive active organic nutrient substitute, along with other additives such as magnesium and iron (Zhang et al. 2015a; Zhi et al. 2020).

The result in Table 2 shows that all three additives effectively improve the removal efficiency of COD, but the performance of yeast extract is more pronounced. As far as the composition of the yeast extract is concerned, it contains nitrogen (amino acids) and some minerals (copper and iron). The presence of these compounds may influence COD reduction. The enhanced COD removal of up to 96.7% was obtained when the concentration of yeast extract was increased to 400 mg/L (Table 3).

**Table 2:** COD removal efficiency with three different additives (yeast extract, magnesium and iron) with PSB cells with time duration in wastewater treatment (Sasaki et al. 2012; Wen et al. 2016)

Time	Yeast extract COD (mg/L)	Magnesium COD (mg/L)	Iron COD (mg/L)
0	~2300	~2200	~2200
24	~1750	~1550	~1650
48	~950	~1070	~1000
72	~500	~800	~700

**Table 3:** The outcome of different yeast concentrations on removal effectiveness of chemical oxygen demand with time duration (Dikshit and Moholkar 2016; Meng et al. 2018)

Time (h)	Yeast concentration 50 mg/L	Yeast concentration 100 mg/L	Yeast concentration 250 mg/L	Yeast concentration 400 mg/L
0	~2250	~2270	~2300	~2450
24	~1750	~1750	~1470	~1510
48	1100	~930	~580	~600
72	~700	~500	~150	~80

As stated earlier, the main benefit of using PSB for wastewater handling is that it can produce an interesting amount of PSB biomass, which is a rich source of protein along with other biopolymers. With these abilities, researchers studied the composition of produced biomass growth and its valuable components.

The main breakthrough in this field is that PSB worked in the treatment of the brewery waste stream. It was also able to produce highly valued PSB cells with the dischargeable COD value of the final stream, which is a value that conforms to the national release value so that there is no need for any post-treatment. The *Rhodopseudomonas* strain is the most efficient one. The obtained PSB cells were a rich source of the desired protein along with other biopolymers such as polysaccharides, carotenoids and coenzyme Q10. The concentration of the PSB cell protein increased to approx. 420.9 mg/g after the reaction (Qi et al. 2017).

### Protein recovery from plant biomass

Dotsenko and Lange investigated the recovery of protein from two different sources: white clover and ryegrass screw pulps. They applied the extraction technique in the presence of aqueous media, also with enzymes such as carbohydrases and proteases to promote extraction behaviour (Dotsenko and Lange 2017). To date, protein extraction from biomass pulp has been unexplored, and more focus has been centred on protein recovery from leaves, primarily by mechanical disintegration. There is a need to upgrade the process of protein recovery from leaves and the pulp. The pioneer of the extraction of proteins from leaves is Pirie, who recommended the fragmentation of fresh green biomass mechanically, leading to separation of the protein from the obtained juice of biomass (Fiorentini and Galoppini 1983).

Several physical and chemical techniques for the separation and purification of protein from the leaves of plants were proposed, but the most relevant methods used for the separation of protein from cellulose-containing biomass are via alkali (Zhang et al. 2014a), aqueous ammonia extraction, and mechanical disintegration of biomass. The main hypothesis of the work done by (Dotsenko and Lange 2017) is that, after screw-pressing the green leaves, a significant proportion of the protein content is left over in the pulp portion, and that protein can be used to feed animals in different ways. The pulp part of green leaves can be used to feed cows, and with the hydrolysis process it can be used as additives in the preparation of food for other animals such as pigs, chickens and fish. The results obtained from their work showed that, by applying the extraction at pH 8.0 on the pulp portion of the leaves, roughly 40% of the total pulp protein was improved and 80% of the protein was restored by proteases (*Savinase 16.0 L, Novozymes*), depending on the dose of this enzyme. The action of enzyme carbohydrases (*Cellic CTec2 and Cellic HTec2, Novozymes*) on pulp hydrolysis did not yield any significant production of protein (Kinsella 1988).

### Protein recovery by adsorption

To date, researchers have been focusing on the development of efficient adsorbents for the immobilization of protein, as there is a crucial need for the fruitful implementation of its applications in different areas. The selection of an appropriate adsorbent for the adsorption of protein depends on several factors, including surface area and large pore sizes with a volume ratio that can be placed on proteins and enzymes with ease (Han et al. 2007; He and Shi 2011; Hartmann and Kostrov 2013; Mohammad 2013; Gascón et al. 2014; Masuda et al. 2014; Deka et al. 2015; Tu et al. 2016). Numerous categories of mesoporous silica substances including MCM-41 (Salis et al. 2009), SBA-15 (Washmon-Kriel et al. 2000), MCF and FMS (Kim et al. 2005) have been effectively used for the immobilization of different substances, e.g. proteins and enzymes (enzymes are mostly protein in nature) as supports or base material.

The biggest drawback in the proper implementation of these porous materials is the restriction in the distribution and movements of adsorbed substances due to the small pore sizes and the inner two-dimensional structural design of these pores. Because of the two-dimensional architecture of the pore size, the researcher proposed new mesoporous silica materials as an adsorbent for proteins. These materials are responsible for exclusive pore availability for adsorbates (proteins and enzymes) due to their interrelated pore assemblies, so they efficiently improve adsorption ability. Still, the practical application of these materials in the immobilization of biomolecules brings complications, as there is a hindrance linked to the mesoporous silica due to its electronically neutral silica skeleton. However, the efficiency of silica-based adsorbents can be enhanced by the surface modification of this mesoporous silica by setting organic functional groups such as the carboxylic (-COOH), phenyl, vinyl, and amine groups for the formation of active sites for the adsorbates (protein/enzymes) (Ramasamy et al. 2019). The introduction of these organic groups on the plane of mesoporous material can efficiently minimize the leaching effect of the adsorbed enzymes (Chong et al. 2004; Maria Chong and Zhao 2004; Wang et al. 2006; Sae-ung and Boonamnuayvitaya 2008; Johari et al. 2014).

A recent study led to the manufacture of silica-based mesoporous Santa Barbara Amorphous (SBA-1) type 1 nanoparticles, which possess a distinctive pattern and surface characteristics. Their practice as grounds for the immobilization of papain is described. In this study, different pore sizes of these mesoporous silica particles were synthesized using the carboxylic functional group by means of co-condensation (Lin et al. 2017). Carboxyethylsilanetriol sodium salt (CES) was added for the integration of carboxyl functional groups as a functional moiety.

The existence of carboxylic functional groups in the mesoporous silica SBA-1 template was validated by an FTIR analysis and C^13^ solid-state NMR spectroscopy (Saikia et al. 2019a). The prepared mesoporous silica nanoparticles were employed as support for the immobilization experiments of papain protein. The obtained results clearly show that large-pore nanoparticles with carboxylic functionality are optimal for the immobilization of the desired protein at the higher end. The major factor behind this immobilization is probably the development of electrostatic interaction between the desired protein and function groups introduced at the surface of the mesoporous nanoparticles. It was reported that modified adsorbent can remove selected proteins from the mixture of protein according to their isoelectric points (Saikia et al. 2019a).

There is great attention towards the extraction, separation, and purification of proteins from composite biological mixtures. In this regard, several methodologies, such as affinity chromatography, precipitation, extraction, and solid-phase extraction, have been developed to segregate the chosen proteins from biological systems (McDonald et al. 2009).

As mentioned in the above example, silica-based support (Iftekhar et al. 2017) with the carboxylic functional group provides proof of the separation of protein from the mixture. It was found that amino acid can also act as an appropriate and efficient ligand in building the networking among proteins or peptides because of the existence of additional functional groups including amine, carboxyl, and tryptophan (Qiao et al. 2018). The researcher tried to incorporate the tryptophan into the silica nanoparticles used as support using cross-linking (Beena et al. 1994). Tryptophan is a hydrophobic amino acid because it consists of an aromatic ring in its side chain, which provides supplementary motive power, primarily pi- pi interactions that help in protein adsorption. They are used to concentrate on Ova proteins that are present in albumin in chickens. The adsorption behaviour of this protein onto the complex silica nanoparticles with tryptophan as a functional group was observed at different pHs ranging from 3 to 8. The results obtained showed that the adsorption of the desired protein increases by up to 1240.3 mg/g compared to its adsorption on unmodified silica nanoparticles, which is 727.6 mg/g. The adsorption operation of the complex material is accredited to the interaction of both pi-pi and hydrogen bonding between the desired protein moiety and adsorbent (Qiao et al. 2018). Table 4 is about the studies presented in the state of the art about the types of adsorbents used for the recovery of specific proteins at given parameters.

**Table 4:** Protein recovery employing immobilization or adsorption on different supports

No.	Adsorbent	Protein	pH	Temperature (°C)	Time (mins)	Adsorption capacity (mg/g)	Reference
1	Tryptophan modified aminated mesoporous silica nanoparticles	Ova Protein	5.0	ambient temperature	25	1240.3 (96%)	(Qiao et al. 2018)
2	Silica (mesoporous) nanoparticles (MSNs) with the SBA-1 moiety, functional group (-COOH)	Papain	8.2	ambient temperature	1600	1138	(Saikia et al. 2019b)
3	Cage-type cubic mesoporous silica functionalized with (-COOH)	Lysozyme	9.6	37	4800	895	(Deka et al. 2015)
4	Mesoporous silica nanoparticles (MSNs)	Haemoglobin	7.4	ambient temperature	20	747.5	(Tu et al. 2016)
5	Mesoporous silica nanoparticles (MSNs) functionalized by 3-aminopropyltriethoxysilane	Catalase	–	ambient temperature	20	~840	(Tu et al. 2016)
6	SBA-15	Bovine serum albumin	4.8	30	–	482	(Maria Chong and Zhao 2004)
7	SBA-15	Lysozyme	10.6	ambient	240	636	(Ma et al. 2017)
8	Mesoporous silica materials (pore size 17.6 nm)	Cellulose	5.0	50	600	410	(Kim et al. 2005)

### Other methods for recovery of protein from different waste sources

Different studies have been done in the past for the extraction of valuable protein products from different wastes (Table 5). For example, the recycling of meat waste has a significant impact on our environment. The study proposed by (Ghosh et al. 2019) includes the use of electric pulses of different voltages and time duration for the chemical extraction of protein present in the wasted chicken breast muscles. The extracted protein from the waste breast muscle exhibits the antioxidant properties as suggested by the Silico analysis (Ghosh et al. 2019). Other studies indicate the recovery of several types of peptides and proteins from the food waste using the subcritical water hydrolysis (SWH) method (Marcet et al. 2016). For example, the application of the sub-critical hydrolysis process using specific parameters, such as type of reactors, optimum temperature, and reaction time waste obtained from fish entrails, recovered 137 mg/g of dry fish (Kang et al. 2001). In other studies, the application of SWH on scallop viscera waste leads to the production of important high molecular compounds as well as amino acids (Tavakoli and Yoshida 2006). In the aqueous phase, the best yield of the simplest amino acid comes from glycine (Kaspar and Reichert 2013). The recovery of 91% protein named Astaxanthin found in shrimp shell waste by enzymatic conversion was performed by (Deng et al. 2020) along with the recovery of other by-products such as chitin. The recovery of collagen protein powder separated from chromium leather scrap waste was studied, revealed to be containing different amino acids and displaying a low concentration of mineral salt that can be used as fertilizer (Dang et al. 2019). A huge amount of waste is produced by the dairy industry, such as whey, the main by-product that is produced during the production of cheese from milk, which is also an abundant source of valuable proteins (Gopinatha Kurup et al. 2019; Tham et al. 2019). The combination process of ultrafiltration and nanofiltration membranes has been used to obtain up to 90% of proteins from whey waste along with lactose under defined parameters (Das et al. 2016).

**Table 5:** Different waste sources for recovery of protein using different methods

No.	Waste source	Types of protein	Method of recovery	% Recovery	Advantages	Disadvantages	Reference
1	Expired dairy products	Milk protein	Liquid biphasic flotation (LBF) method	~94	Waste reduction, environmental benefits	Recycling is needed as the use of alcohol and high salt conc.^1^	(Tham et al. 2019)
2	Slaughterhouse blood	Haemoglobin peptides	Enzymes and high hydrostatic pressures (HHPs)	~84 peptides yield	Antioxidant and functional properties, cheaper and simpler method	–	(Álvarez et al. 2012; Marcet et al. 2016)
3	Waste from the poultry process	Amino acid	Subcritical water technology	~11.4	High production yield along with energy	Chemical usage	(Zhu et al. 2010)
4	Waste activated sludge	Proteins from sludge flocs	Thermal alkali hydrolysis (TAH)	~67.5	High yield of solubilized protein is obtained	Some protein loss in the form of ammonium and nitrates	(Song et al. 2019)
5	Rohu fish waste	Protein from fish waste source	pH Shift Method	31.8 via acid method/ 31.1 via the alkaline method	Isolates via alkaline methods were more rigid	Protein with good yield and functionality after recovery	(Surasani et al. 2017)

^1^conc. concentration

## Protein recovery by membrane technology

Membrane technology has been utilized and commercialized to regain valuable substances including proteins from different wastewaters such as potato processing wastewater (PPW) (Dabestani et al. 2017), poultry processing wastewater (Lo et al. 1997), alfalfa wastewater. and dairy waste streams. Different membrane processes including microfiltration (MF), ultrafiltration (UF), and reverse osmosis (RO) have been extensively applied in dairy-producing companies to isolate different components such as casein and proteins from their waste streams (Harmen J.Zwijnenberg et al. 2002).

### Protein recovery from wastewater of the potato processing industry by membrane technology

Patatin is the main protein of potato wastewater and well known for its good functionality. The protein possesses many important features, including a molecular weight of around 40–45 kDa along with an amino acid index (EAAI) of about 89%, which is comparatively high compared to that of many other proteins present in plants and animals (Pouvreau et al. 2001; Strætkvern and Schwarz 2012).

Traditionally, there has been a diverse list of techniques for obtaining this valuable protein from potato fruit juice. The most applied techniques were concentration, coagulation with heat, precipitation, ion-exchange chromatography, and ion exchange using Expanded Bed Adsorption (EBA), and many more techniques were used to restore protein from potato fruit juice (PFJ). These approaches have been proven to obtain a high yield percentage of protein, but some processes, such as the application of heat and a harsh environment like acid or alkali, have been unsuccessful in recovering completely intact (non-denatured) high-quality protein (Løkra et al. 2008).

**Fig. 1:**
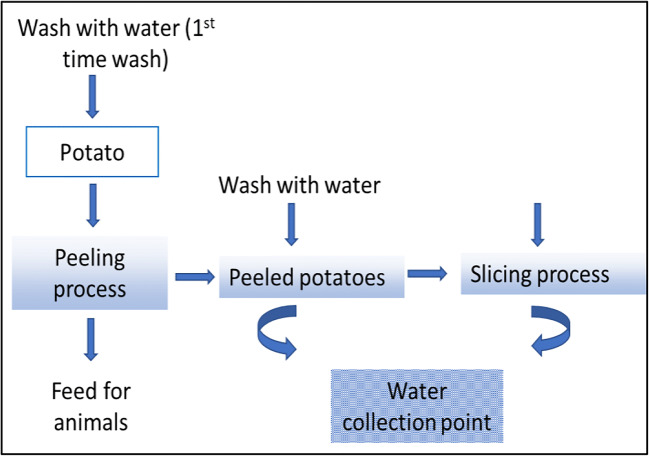
presents potato chips production and point of wastewater collection (Dabestani et al. 2017; Fritsch et al. 2017)

**Fig. 2:**
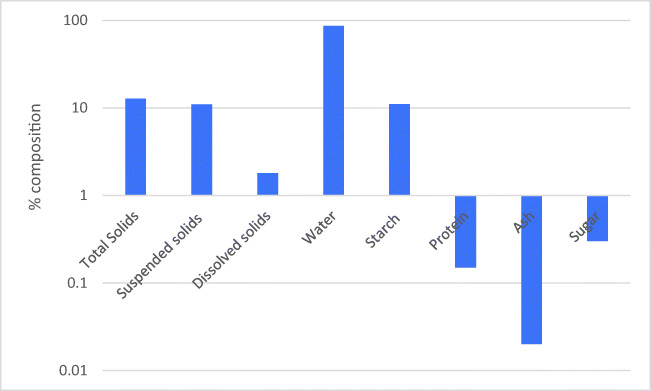
The composition of potato process wastewater (Mishra and Arora 2004; Guo et al. 2018)

Figure 1 shows the PPW production and collection point, while Fig. 2 illustrates the composition of the process water collected from the source (Dabestani et al. 2017). Several combinations of pre-treatments, such as centrifugation (with specifications; time 20 min at 8000 g and 20 °C), sedimentation for 4 h followed by filtration using filter paper with specifications of 2.0, 2.5, and 0.22 μm and PVDF/MF, have been used before applying the ultrafiltration process.

The strategic process with an explanation of the purposes for applying each pre-treatment and treatment step on wastewater is shown in Table 6. It was demonstrated that transmembrane pressure increases suddenly due to the phenomenon known as concentration polarization, and after that the feed was only pre-treated by centrifugation. After some time, there was no significant increase, but this may be due to the deposition of biopolymers, especially proteins, on the surface of the membrane. The increasing concentration of protein in the retentate of membrane filtration over time leads to the cake layer formation and pore blocking process. However, the pre-treatment of feed by centrifugation leading to microfiltration improved membrane performance concerning the fouling.

**Table 6:** Schematic representation of pre-treatment of PPW to reach the existing separation efficiency (Rajewska and Janiszewska 2017)

The waste stream from the potato processing industry	
Pre-treatment techniques	Effects of pre-treatment
Effect of centrifugation	Efficient for removing large particles such as starch and fibres from wastewater
Filtration by the paper filter (pore size 2.5 μm)	Effective removal of insoluble and soluble starch and fibre from wastewater
Filtration 0.22 μm PVDF microfiltration	Removal of substances with a size in the micrometre range
Ultrafiltration by polysulfone membrane	The retentate of membrane filtration is rich in the desired protein

The recovery of protein using ultrafiltration along with the obtained concentration of the desired product was determined by applying an assay known as the BCA test. The initial calculated concentration of protein in the feed solution after applying centrifugation was 1.55 ± 0.03 g/L. After applying a different pre-treatment process on the feed or wastewater, the amount of protein was determined in the obtained permeate of each process. The recovery percentage of protein was 72% by applying centrifugation to the feed solution, and this value fell to 62% after several pre-treatment applications (Table 7). However, it is also clear from Table 5 that the concentration of protein is increased in the retentate of the experiment including centrifugation, followed by MF with filter paper with a specification of 2.5 μm for 20 h.

**Table 7:** Protein rejection by ultrafiltration membrane is calculated based on the bicinchoninic acid assay (BCA assay) (Xu et al. 2016; Li et al. 2020)

No. of Experiments	Protein recovery (%)	Concentration factor (CF)
Centrifugation	72	2.2
Sedimentation (Sed.)/cent.	70	2.2
Centrifugation/microfiltration (MF)	67	1.9
Centrifugation/filter paper 20 μm	63	1.8
Centrifugation/filter paper 2.5 μm	57	2.1
Filter paper 2.5 μm/MF	N/A	N/A
Centrifugation/filter paper 2.5 μm/ MF	62	2.3
Cent./filter paper 2.5 μm/ MF (long-term 20 h)	62	3.5

In each stage of the process, SDS-Page was applied to understand protein bands with dissimilar molecular weights. The results confirmed from the obtained bands of proteins with bigger molecular weight constitute the desired potato protein (patatin family), and other proteins remain intact during the sequence of several treatment stages (Li et al. 2019b). The obtained proteins remained intact from the denaturation process and no separate band was observable in the permeate of the UF membrane (Rajewska and Janiszewska 2017). The concentration of protein in each experiment was determined by examining the filtrate of filter paper of 2.5 μm (MF feed), permeate of MF and permeate of UF using the LC-OCD technique (Miedzianka et al. 2012).

The total DOC present in the feed of the MF membrane was 2306.21 mg/L with 6% of DOC containing biopolymers, therefore amounting to about 142.05 mg/L. After MF filtration, contents such as DOC and biopolymers in the permeate of MF that would be the feed for ultrafiltration were reduced to 1988.51 and 104.39 mg/L, respectively (5.25% of the dissolved organic carbon was biopolymers). The amount of protein is 100% in the feed of the ultrafiltration membrane. After filtration, the concentration of protein present in the UF permeate was negligible, which indicates that approximately all the protein present in the feed of UF revealed by LC-OCD was removed in the retentate. It was concluded from the work done by the researcher that the desired protein from the PPW can be accumulated up to 3.5 times that of its initial concentration using an efficient membrane. The polyethersulfone UF membrane with an MWCO of 10 kDa was proven to be efficient in concentrating the protein from potato waste streams (Dabestani et al. 2017). However, loss of protein was unavoidable when multiple pre-treatments were conducted. To avoid the fouling mechanism of the membrane, several pre-treatments were applied, such as the addition of chemicals (acid or basic) to lessen the denaturation of the desired protein. Membrane technology for protein recovery from potato wastewater has proven to be efficient, but it is still important that the value of the obtained protein in terms of its quality and purity be understood. Furthermore, it is also desirable to study the effect and concentration of total glycoalkaloids (TGA), a natural toxin already existing in potato and potato harvests, and attention should be paid to regulating the accumulation of this toxin in the obtained protein to evaluate the quality of the final product for future applications.

### Protein recovery from wastewater of the poultry processing industry by membrane technology

With the rapid increase in the human population, there has been an immense rise in meat intake. The daily consumption of poultry (especially chicken) is growing at an even higher rate than that of other popular meats such as beef or pork (Castro-Muñoz and Ruby-Figueroa 2019). In this current situation, the waste streams from the poultry industry such as carcass debris and body fluids, mainly blood, are the main impurities, along with fat. Blood and debris are rich in proteins. Wastewaters from poultry have a higher biological and chemical oxygen demand than normal sewage waste streams because of the high concentration of proteins. A method was proposed to restore valuable protein from poultry-handling industry waste streams by applying membrane technology such as the ultrafiltration process. In their study, researchers explored the possibility of retrieving protein from poultry wastewater via UF with an MWCO of around 30,000 kDa along with the development of parameters for the effective performance of the total process (Lo et al. 2005). In this perspective, if the ideal process circumstances are recognized, it is extremely relevant that UF can function at peak flux in the desired period, thus refuting the contrary results produced by the contaminated membrane (Le Roux and Belyea 1999).

It is well-known that UF has been extensively applied to the separation, concentration, and refinement of colloidal and higher molecular weight constituents present in solutions (Lo et al. 1997). Most fats in poultry streams were removed from wastewater through the implementation of different primary pre-treatments, including dissolved air flotation (DAF), and the placement of a UF unit right after the physical treatment of wastewater could greatly decrease membrane fouling. There is a need to study the methods to recover membrane performance by applying some special cleaning procedures to make this process effective for cost and durability. There is also a need to minimize the concentration of nutrients in the effluent for downstream processing (separation, concentration, and purification) to maintain the expected quality and functionality of the desired protein. The non-thermal UF process keeps protein denaturation from suffering during thermal procedures.

**Fig. 3:**
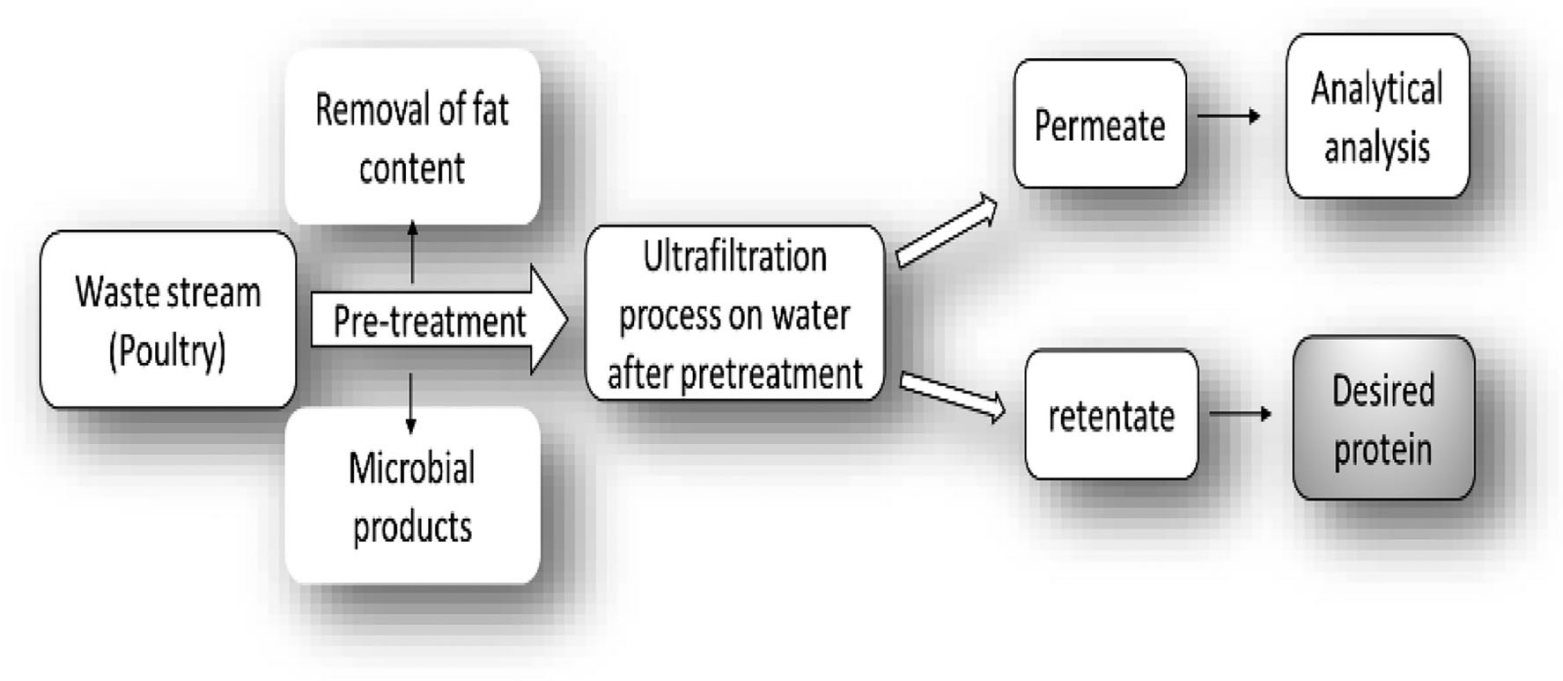
Ultrafiltration membrane process for recovery of desired protein from poultry processing wastewater (Lo et al. 2005)

A diagrammatic presentation of procedures using a polysulfone UF membrane with a molecular weight cut-off of 30,000 Da is shown in Fig. 3 for the recovery of the desired protein from the poultry processing wastewater in the retentate after the membrane process. The wastewater undergoes the pretreatment to remove the fats and other components before passing this water from the membranes (to avoid membrane clogging). The polymer membrane, especially one made of polysulfone, is a commonly used UF membrane in the food sector. The polysulfone membrane has been recognized as having the least fouling drifts when treating skimmed milk (Kumar et al. 2013).

A flat-sheet compartment with a membrane with a specific area of 30 cm^2^ was used for the process assessment. The total fat in the waste samples was observed through the partition-gravimetric (PG) method (EPA Methods 5520 B).

A distinctive flux report and variation in protein concentration through the membrane process are shown in Table 8 above. It can be predicted from the table that rapid flux decline takes place within just 20 min of filtration and continues to decrease throughout the process. Along with a decrease in the flux of the membrane over time, the protein content increased from 80 to 273 mg/L (Lo et al. 1997).

**Table 8:** Changes in the flux of the membrane along with the concentration of desired protein during the ultrafiltration of poultry wastewater at specified parameters 25 °C; pH (neutral); transmembrane pressure of 14 psi (Kumar et al. 2013)

Time (mins)	Membrane flux (Lm^−2^/h)	Protein contents (mg/L)
0	264	80
20	140	~90
40	~138	100
60	130	~150
80	~128	200
100	125	273

UF treatment can decrease the COD in the main waste stream by 58.86% (Lo et al. 2005). A negligible amount of protein was found in the permeate of the membrane. Residual COD in the permeate was normally documented with a safe direct discharge value (200 mg/L). This high COD value of permeate is due to the use of chemicals such as cleaners, antiseptics and flocculants throughout the processing, especially in pre-treatment, and there is possibly residual waste which causes a high COD value. The results obtained are in keeping with a prior report that elaborated on the UF process for the removal of sericin from wastewater from the silk degumming industry (Fabiani et al. 1996). The procedure produced permeate with high COD (800 mg/L). To overcome this high value, reverse osmosis was used, after which the COD value fell as low as 50 mg/L. Consequently, after ultrafiltration, the ecological effects of final discharge need to be assessed in greater detail before it can be emitted into rivers and oceans, for example.

**Fig. 4:**
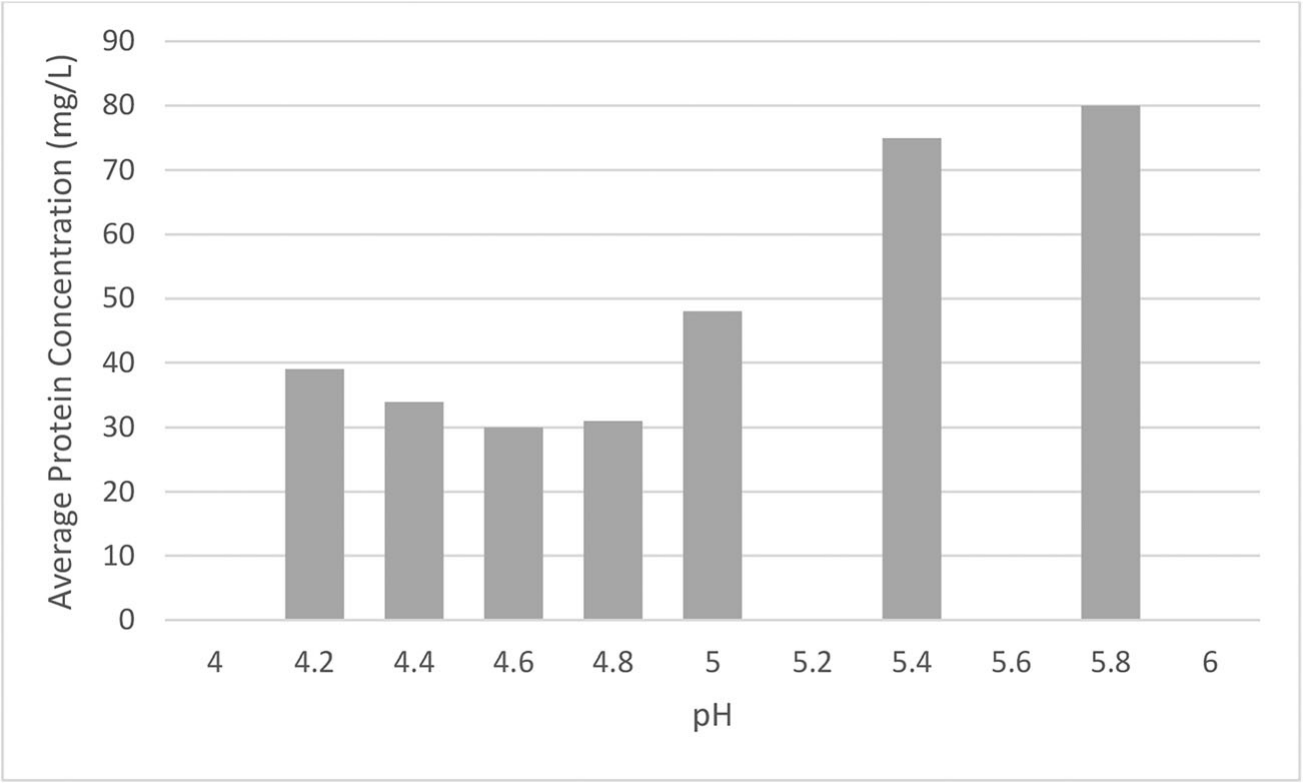
Estimation of the isoelectric point of protein restored by fractionation method (Lo et al. 2005)

It was shown by (Martínez et al. 2000) that pH is a highly significant factor and has an obvious influence on the configuration of protein molecules. The properties, including the electrical interaction of protein molecules, play a significant part in defining their interfaces with the surface of the membrane. Hence, for additional evaluation related to the outcome of pH on the membrane filtration process, an isoelectric point (pI) of protein removed from the waste stream (poultry) was investigated using the fractionation method (Fig. 4). It is vital to consider the optimum pH value of the solution and isoelectric point, which can prevent the formation of the cluster due to agglomeration (Honda et al. 1993; Guinee et al. 1997).

The isoelectric point of protein present in the waste stream was located at around pH 4.6 (Fig. 4). The isoelectric point of the desired protein was at pH 4.6, which is far from the optimum pH value of 6.74 (Lo et al. 2005). pH 6.74 is favourable for the ultrafiltration of poultry wastewater, as that pH is effective for the prevention of protein coagulation that could cause adverse fouling and affect the overall membrane process. Even though extreme membrane fouling was still unavoidable after pre-treatment, back-flushing, or sampling flushing of the fouled membrane with chemical cleaning, was efficient in regaining the working performance of the membrane. An integrated approach to recovering protein from the poultry waste stream, including microfiltration, ultrafiltration, and vacuum membrane distillation, was studied (Honda et al. 1993).

The researcher tried to restore the desired protein in water according to the filtration sequence already used with membranes with an MWCO between 3 and 30 kDa. The membrane that was chosen for the next step is based on the removal of the COD value along with the protein retention capacity in the retentate. The integrated membrane process, including MF, UF, and vacuum membrane distillation (VMD), was used to purify water and protein.

In the case of the last stated procedures, practically all the protein obtained in the feed that is the permeate of the ultrafiltration process can be restored in the retentate. The solution proposed, using an integrated process, allows an almost nine-fold decrease in the capacity of waste produced. This method also gave the outlook to use the recovered protein concentrate as a feed and for food production (Honda et al. 1993).

In the state-of-the-art work performed by (Lo et al. 2005), the concentration of the protein is about 40% of the total solids (TS) in the retentate. Direct evaporation could be helpful for the recovery of solids present in the concentrated final product. About 70% of the dry solid weight was recovered this way. By the application of the ultrafiltration process (polysulfone membrane) with an MWCO value of 30,000 Da, proteins from the poultry waste stream are concentrated in the final retentate, followed by a reduction in the total COD value applicable for discharge (Iwuoha and Umunnakwe 1997). It was proposed that, if other nutrient loads were reduced in the effluent undergoing downstream processing, one can avoid any impurity in the desired recovered protein and prevent the protein from harsh thermal degradation effects (Lo et al. 1997; Le Roux and Belyea 1999). Researchers also tried to recover protein by applying ultrafiltration on the solution obtained after mechanical extraction to debone the turkey residue. The ultrafiltration on that solution was done without prominent fouling (clogging) of the membrane during the procedure.

### Protein recovery from alfalfa-processing wastewater using membrane technology

Leaf protein obtained from alfalfa juice is a vital protein for animal feed and human food. Previously, the researcher recognized alfalfa juice as a competent source of good-quality leaf proteins. The main significant features of this protein are that they are abundant in nature with a high concentration of protein, valuable nutritional aspects and most importantly no animal cholesterol. Just like other food processing industries, the alfalfa processing industry produces plenty of wastewater. This wastewater is rich in protein, containing up to 50% of hydrophobic proteins. The wastewater contains diluted alfalfa juice along with cleaning detergents (Zhang et al. 2015b). Another researcher also recognized the wastewater of alfalfa as a source for nutrient renewal applicable to irrigation and restoring biomolecules (Lamsal et al. 2007; Xie et al. 2008).

Different separation systems were used for the removal of alfalfa proteins from diverse sources. The most commonly applied methods are chromatography (Ibarra-Herrera et al. 2011) fractionation by means of solvents (Koshchuh et al. 2004), heating (Bals and Dale 2011), crystallization (Firdaous et al. 2010), molecular sieve chromatography, centrifugation (Arulvasu et al. 2014), and ion exchange technology (Ibarra-Herrera et al. 2011). However, none of these methods provide good removal/separation efficiency for protein.

**Fig. 5:**
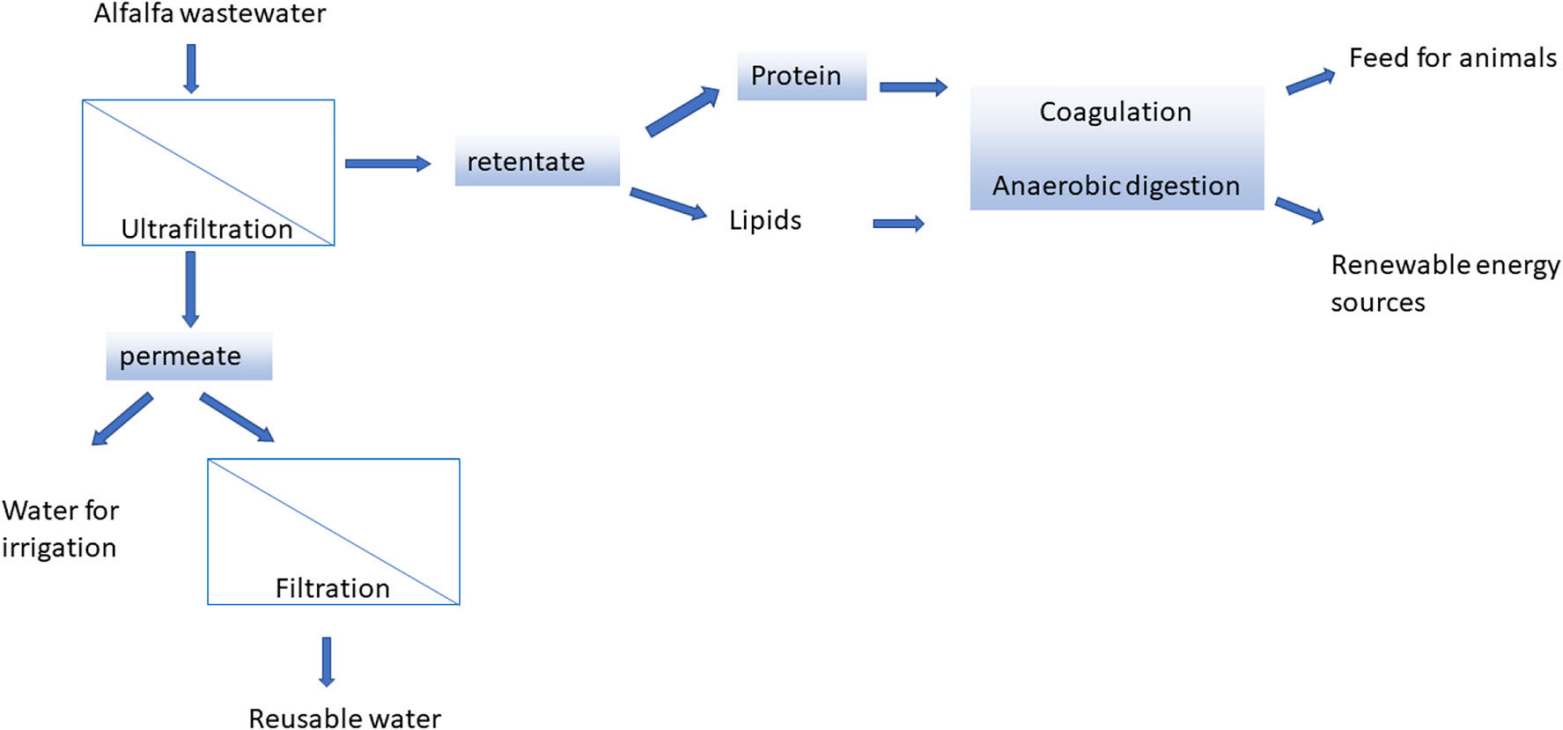
Schematic representation of membrane process on the alfalfa waste stream and its applications (Zhang et al. 2015b; Gao et al. 2018)

The ultrafiltration (UF) process shown in Fig. 5 can facilitate the downstream processing of protein present in alfalfa juice, whereas the concentration of the filtration process contains a protein that can be separated using precipitation for the production of feed for animals (Zhang et al. 2015a). It can also be effective for the production of renewable energy followed by anaerobic digestion (Venkata Mohan et al. 2008). There is also the possibility that permeate obtained after UF can undergo an additional filtration through a membrane of smaller pore size (nanofiltration or reverse osmosis), which results in drinking water (Sarkar et al. 2006), and the water can also be used to irrigate fields.

In the filtration process of alfalfa wastewater using the UF membrane, the filtration efficiency decreases over time because membrane fouling leads to an effect known as concentration polarization (Ma et al. 2017). If this effect continues, the efficiency of the membrane process faces a severe decline and results in increases in the overall procedure cost. To overcome this problem, a new investigation including a rotating disk membrane (RDM) unit was applied to lessen membrane fouling and recover flux performance. In the RDM process, the main phenomena that overcome the concentration polarization effect increase the shear rate (Zhang et al. 2014b).

To understand the recovery of protein along with the behaviour of membrane fouling during this process, (Zhang et al. 2014b) made efforts to accomplish a four-factor and three-level central combined response surface methodology (CCRSM) experimental plan to understand the collective operation parameters, including the significance of transmembrane pressure (TMP), feed flow rate, the effect of shear, and temperature. The ideal operation settings are likely to qualify the probable implementation of RDM for membrane wastewater behaviour in the future (Bensadallah et al. 2016).

Table 9 illustrates COD rejection, the removal efficiency of protein, and flux value of membranes, along with fouling, permeability recovery after membrane cleaning, and cost behaviour of processed UF membranes. The PES50 membrane showed the smallest removal of COD, rejection of protein, and maximum flux, probably due to the larger pore size distribution. However, the average diameter of protein present in the feed solution (60–90 nm) is bigger than that of the membrane PES50 having a pore size of 10 nm. Therefore, according to the concept, most leaf proteins should be retained by the membrane (Zhang et al. 2015b).

**Table 9:** The distinctive parameters of permeate for several membranes at a given temperature and pressure (Zhang et al. 2017, 2019)

Used membrane	Surface material	MWCO (kD)	^1^Water permeability	COD removal efficiency %	Protein removal %	Fouling	^4^Flux	Recover permeability %	Energy cost (kWhm^−3^)
Polyethersulfone20	^2^PES	20	>30–40	31.52	72.92	1.6051E+10	83.6	70.57	297.84
UH030P	^3^PESH	30	40-50	27.46	70.83	7.0617E+09	153.0	80.07	162.74
PES50	PES	50	>70	25.42	66.67	7.0639E+09	183.6	73.91	135.62

^1^Water permeability (L m^−2^ h^−1^ bar^−1^)

^2^*PES*, polyethersulfone

^3^*PESH*, permanently hydrophilic polyethersulfone

^4^Flux (L/ (m^2^ h))

### Protein recovery from the dairy waste stream using membranes

Dairy wastewater is typified by severe pH changes and typically contains high concentrations of organic matter, solids and nutrients, as well as dissolved inorganic pollutants and traces of cleaning agents (de Souza Santana et al. 2016). In dairies, membrane-based technologies, and their combination with other methods such as biological, chemical and physical ones, are likely to be used for water recycling and resource harvesting (de Souza Santana et al. 2016).

The study performed by (Bortoluzzi et al. 2017) demonstrated that dairy wastewater handled by membrane-integrated processes can possibly meet the environmental disposal requirements of treated wastewater in the collection of water, as well as supporting the reuse of water in the dairy plant itself as well as in cooling and heating procedures. In the above-stated study, two stages of the integrated membrane filtration system have been used. The integration system uses the MF + NF process in one stage and the MF + RO system in other stages, using different operation parameters while treating the dairy processing waste stream. The results obtained from this study indicate the removal of suspended solids to a large extent using the MF membrane along with the reduction of turbidity and colour. The combination of the MF + NF process is more effective in the reduction of turbidity up to 96%. Moreover, the complete reduction of turbidity (100%) along with colour reduction (100%) as well as TOC removal of 84% was observed using the MF + RO integrating system (Bortoluzzi et al. 2017; Sert et al. 2017).

In state of the art, the use of UF membranes concerning their pore size and surface charge for the fractionation of the dairy components is accepted as a promising process especially in the valorisation of whey protein. However, due to the presence of a large number of proteins such as bovine serum albumin, ᾰ- and ß-lactoglobulin in the dairy wastewater can limit the use of the UF membrane, which contributes towards the severe membrane fouling (Barukčić et al. 2015; Brião et al. 2017). In a recent study (Damar et al. 2020), the whey protein recovery was investigated using different commercially available UF membranes with different characteristics. The membranes used in this study were regenerated cellulose acetate membrane, composite fluoropolymer membrane and polyethersulfone membrane. All the used membranes have the same cut-off of 10 kDa. The high roughness of the composite fluoropolymer membrane more likely increased the rejection of lactose and whey proteins in the retentate. The results indicated that the surface hydrophobicity of composite fluoropolymer and polyethersulfone membrane are considerably involved with the fouling resistance. Despite the low selectivity, the regenerated cellulose acetate membrane presented a better competence for the concentration of whey proteins due to its high antifouling content. Table 10 presents the membranes used for the recovery of proteins from different wastewaters and the limitation in their usage at a larger scale.

**Table 10:** Membranes used for the recovery of protein from waste sources and their limitation of the application

No.	Waste source of protein	Membrane specification	Limitations	Reference
1	Yellow fin tuna (Thunnus albacores) viscera	UF-regenerated cellulose (RC) membranes (76 mm in diameter)	Membrane fouling with higher molecular weight compounds	(Pezeshk et al. 2019)
2	Anti-scorpion serum production wastes	UF (10 kDa) and electrodialysis process	Multiple rinsing of the membrane was essential	(Bensadallah et al. 2016; Castro-Muñoz and Ruby-Figueroa 2019)
3	Tuna processing by-products	Cascade integrating UF (n.r.) and NF (n.r.) membranes	Membrane clogging due to the high protein contents	(Klomklao and Benjakul 2017)
4	Tilapia by-product protein hydrolysate (TBH) separation	Flat-sheet RC with MWCO of 10 and 5 kDa	n.r.	(Roslan et al. 2017)
5	Dairy waste in the form of whey proteins	UF (10 kDa). membrane pore size 200–250 nm	Whey protein (b-lactoglobulin) aggregation leads to severe fouling	(Steinhauer et al. 2015; Ganju and Gogate 2017)
6	Whey, a by-product of cheese	Flat-sheet membrane (0.0061 m^2^) and a spiral membrane (0.22 m^2^)	Aggregation of b-lactoglobulin protein takes place as membrane shows retention of this moiety	(Baldasso et al. 2011; Argenta and Scheer 2020)

n.r. not reported

### Protein recovery from seafood processing wastewater

Over the last decades, the importance of the shrimp industry to global economic development has been evident through shrimp exports and imports and the associated research performance. Wastewater produced in aquaculture needs an urgent management process to reduce its effects on the environment (Ng et al. 2018)**.** Research has recovered valuable components such as proteins from the shrimp wastewater using several methods. In a recent study, the protein-rich biomass from shrimp boiling water (SBW) was treated with different flocculants at different pHs to recover protein using flocculation and sedimentation techniques. The results obtained during this study indicated a maximum sedimentation of up to 80% of proteins from the biomass at pH 4 (Forghani et al. 2020). In another case study, tuna processing industrial wastewater has been subjected to hydrolysis for the purpose of protein recovery using the fractionation process by the UF and NF membrane cascade in a continuous industrial-scale process. The proposed model was applied for the investigation of the most suitable configuration of UF and NF membranes in linear or dual cascades to obtain the maximum recovery of protein from the wastewater (Abejón et al. 2018).

The increase in the consumption of fish because of its high protein content and other nutritious values, especially tuna, leads to an increase in the wastewater produced during the processing in the industry. Several studies are being conducted on the recovery and separation of valuable components from the wastewater coming from the fish processing industry. The work performed includes the valorisation of tuna processing waste biomass for the recovery of valuable proteins and peptides with the integration of enzymatic hydrolysis and the membrane fractionation process (Saidi and Ben Amar 2016). The applied method in this study is important for producing valuable bioactive products from the tuna fish processing wastewater and in lowering the organic content of the wastewater generated from the food processing industry. In this study, the enzymatic hydrolysis coupled with the UF and NF membrane system leads to the biotransformation of protein present in the tuna processing industry wastewater to tuna protein hydrolysate. The obtained results from the evaluation of the amino acid composition and antioxidant study of the recovered protein show the presence of many valuable amino acids (aspartic acid, glycine, alanine, valine and leucine) in the extracted protein (Sayari et al. 2016). All these amino acids are found in the retentate of the NF membrane after membrane fractionation.

## Protein recovery and membrane fouling

Membrane fouling is identified as the irreversible deposition of sediments on the active surface of a membrane, resulting in flux decline during the process and a loss of active operation. This is a problem during the filtration of high organic content wastewater such as industrial water, brackish water, and seawater (Penña et al. 2013; Castro-Muñoz et al. 2019). One of the main obstacles to applying membranes for protein recovery from the targeted wastewater is membrane fouling due to the concentration polarization effect (Castro-Muñoz et al. 2019). The term concentration polarization can be defined as the reversible fouling process, thus reducing the flux and permeability through the membrane (Bhattacharjee et al. 2017). There is also another phenomenon called pore blocking that also occurs during the recovery of proteins and is defined as an irreversible process; the pore blocking also leads to consequences such as flux decline during the membrane filtration process (Bhattacharjee et al. 2017; Issaoui and Limousy 2019). When studying the recovery of protein from high organic content waste streams using MF and NF membranes, the major drawback that results in reduced flux is the concentration polarization effect and pore blocking with cake layer formation on the surface of the membrane over time (Park et al. 2017; Aghapour Aktij et al. 2020).

The filtration of high organic content wastewater through membranes is prone to fouling, and that leads to a significant increase to the cost with regard to the industrial-scale process. Consequently, there is a vital need for the potential cleaning procedure to minimize the membrane fouling for the efficient process and durability of membranes (Humpert et al. 2016). One of the most effective and useful solutions includes the backflushing and rinsing of the membrane after and during the procedure, and the other is chemical cleaning of the membrane after each process (Wallberg et al. 2003; Bogati et al. 2015). In order to reduce the membrane fouling during the process the membranes were rinsed with the permeate of the filtration process, as the permeate possesses the same pH as the original solution. Also, it is more likely that most of the pollutants that stick on the surface of the membrane were soluble to the permeate (Beyer et al. 2017). The other method used for the sustainable operation of the membrane process is chemical cleaning of both UF and NF membranes, using different chemical agents such as sodium hypochlorite (NaClO) (Li et al. 2019a), which is effective for membrane fouling via organic wastewater treatment and microbial fouling (Malczewska and Żak 2019). Table 11 presents the cost analysis of protein recovery using membrane technology from different waste streams.

**Table 11:** Cost analysis of different wastewater treated using membranes

No.	Wastewater	Operational cost	Treatment method	Reference
1	Dairy waste stream (milk protein)	64–3374 US$/cubic metre of treated effluent	Oxygen injection into the physicochemical treatment system	(Martín-Rilo et al. 2015)
2	Microalgae biomass (extraction of protein)	0.12 US$/kg of microalgae sample	Polyethersulfone membrane	(Lorente et al. 2018)
3	Whey protein	US$436,000	UF/RO process	(Wen-qiong et al. 2019)
4	Milk protein	US$322,882.40 (annual cost)	RO operating system	(Brião et al. 2019)

Most of the membrane process plants designed for the purification of wastewater from several sources utilize repeated cleaning of the membranes and their modules, using a range of chemicals to reestablish the membrane performance to the same level as before fouling. As mentioned in Table 11, the chemical cleaning of the membranes has some characteristic drawbacks, and one of the biggest is reducing the cost-effectiveness of the overall process and putting an extra burden on the atmosphere. Hence, it is now time to thoroughly research the innovation of new strategies to overcome traditional remedies. For future studies, the researcher should focus, practice, and prioritize the more mechanical cleaning procedures, such as, e.g. the backflushing of the membrane during and after the procedure, rather than the traditional chemical cleaning method (Bogler et al. 2017; Matin et al. 2019; Nunes et al. 2020).

The recovery of protein from wastewater using several techniques, including membrane technology, will always remain an interesting topic for researchers and scientists all over the world. New advances day by day concerning new membrane materials and the integration of membranes with other processes is a matter of interest when the protein recovery is involved. However, we should explore the proper functional and environmental benchmarks for membrane-technology-assisted protein recovery from a variety of waste streams to overcome the hindrance that lies within the transfer of this process from laboratory-scale studies to the profitable market (Shahid et al. 2020; Xiao and Zhou 2020).

## Conclusion

As discussed, there are several pathways towards the promising recovery of proteins from different waste sources. A variety of wastewaters have been investigated for the efficient recovery of protein using different techniques in the state of the art. It was demonstrated that purple photosynthetic bacteria along with microalgae are the most efficient and dominant mediators for the removal of organic substances and nutrients from red meat, pork- and poultry-processing wastewaters. Microbial protein is proven to be a worthwhile and available protein supply to satisfy the need for human as well as animal diet stability. A simple one-step treatment using bacteria provides both benefits, including the supply of protein sources and treatment of high-COD non-toxic organic wastewater. The process of protein recovery by bacteria can fulfil the requirement to release wastewater streams without any treatment. The obtained bacteria cells contain high concentrations of valuable molecules including proteins, polysaccharides, carotenoids, bacteriochlorophyll and coenzyme Q10. However, there are some drawbacks to this process, including the existence of many nucleic acids in some entities, which makes its utilization by human beings unsuitable, and these molecules should be handled properly and within the limit range. Moreover, post-treatment of the obtained single-cell protein is also significant because of its susceptibility to any impurities. In the coming years, it has been predicted that microorganisms will be an important source of food additives due to their efficiency in manufacturing high-quality protein from waste substances.

However, to cover the future need for protein and achieve self-sufficiency, there is still a gap to developing new technologies for large-scale manufacturing that needs to be overcome. The implementation of membrane technology for protein recovery has also been proven to work effectively. Also, the integration of MF, UF, and VMD can allow a reduction in the volume of waste generated that is almost nine times greater and can establish an option to make a protein concentrate suitable for human and animal needs. The recovery of different types of proteins from wastewaters using membranes has been discussed in detail in this paper. The advantages associated with using membranes in the recovery of proteins along with other valuable products from the potato processing, dairy and seafood processing industries has been taken into special consideration in this study. Membrane fouling is always associated with the process of protein recovery. The backflushing of the membrane with available cleaning reagents was found to be proficient in improving the restoration ability of the membrane. The development of efficient technologies and improvement in traditionally available techniques of protein recovery from waste streams can provide high-quality protein and reduce the gap between the demand for and supply of proteins, making them available for humans and animals. However, there is still a need for more research to find new and more potential wastes as a protein source via the implementation of membranes for the sake of the long-term stability of our system.

## Data Availability

Not applicable.

## Abbreviations

BCA: Bicinchoninic acid assay
CCRSM: Central combined response surface methodology
COD: Chemical oxygen demand
CES: Carboxyethylsilanetriol sodium
CF: Concentration factor
DOC: Dissolve organic carbon
EAAI: Amino acid index
EBA: Expanded bed adsorption
HOBs: Hydrogen-oxidizing bacteria
kDa: Kilodalton
MWCO: Molecular weight cut-off
mg/L: Milligramme per litre
MF: Microfiltration
NH_4_-N: Ammonium
NF: Nanofiltration
nm: Nanometre
PES: Polyethersulfone
PPB: Purple phototrophic bacteria
PSB: Photosynthetic bacteria
PO_4_-P: Phosphate
PFJ: Potato fresh juice protein
PPW: Potato processing wastewater
RO: Reverse osmosis
RDM: Rotating disk membrane
SBA: Santa Barbara Amorphous
SCOD: Soluble chemical oxygen demand
SCP: Single-cell protein
SWH: Subcritical water hydrolysis
TKN: Total Kjeldahl nitrogen
TN: Total nitrogen
TP: Total phosphorus
TMP: Transmembrane pressure
TS: Total solids
UF: Ultrafiltration
VMD: Vacuum membrane distillation
μm: Micrometre
